# Interviews with HIV Experts for Development of a Mobile Health Application in HIV Care—A Qualitative Study

**DOI:** 10.3390/healthcare11152180

**Published:** 2023-08-01

**Authors:** Jannik Schaaf, Timm Weber, Michael von Wagner, Christoph Stephan, Jonathan Carney, Susanne Maria Köhler, Alexander Voigt, Richard Noll, Holger Storf, Angelina Müller

**Affiliations:** 1 Institute of Medical Informatics, Goethe University Frankfurt, University Hospital, 60590 Frankfurt, Germanyrichard.noll@kgu.de (R.N.);; 2 Department of Medical Information Systems and Digitalization, University Hospital Frankfurt, 60590 Frankfurt, Germany; 3 Department of Internal Medicine, Infectious Diseases, University Hospital Frankfurt, 60596 Frankfurt, Germany; 4 Institute of General Practice, Goethe University Frankfurt, 60596 Frankfurt, Germanya.mueller@allgemeinmedizin.uni-frankfurt.de (A.M.)

**Keywords:** HIV infections, AIDS, mHealth, telemedicine, digital health

## Abstract

The Communication and Tracing App HIV (COMTRAC-HIV) project aims to develop a mobile health application for integrated care of HIV patients due to the low availability of those apps in Germany. This study addressed organizational conditions and necessary app functionalities, especially for the care of late diagnosed individuals (late presenters) and those using pre-exposure prophylaxis. We followed a human-centered design approach and interviewed HIV experts in Germany to describe the context of use of the app. The interviews were paraphrased and analyzed with a qualitative content analysis. To define the context of use, user group profiles were defined and tasks derived, which will represent the functionalities of the app. A total of eight experts were included in the study. The results show that the app should include a symptom diary for entering symptoms, side effects, and their intensity. It offers chat/video call functionality for communication with an HIV expert, appointment organization, and sharing findings. The app should also provide medication overview and reminders for medications and appointments. This qualitative study is a first step towards the development of an app for HIV individuals in Germany. Further research includes involving patients in the initial app design and test design usability.

## 1. Introduction

In 2016, more than 36.7 million people were infected with the human immunodeficiency virus (HIV) or suffering from the acquired immunodeficiency syndrome (AIDS), the final stage of the HIV disease [1]. Untreated HIV increases the risk for a wide range of infectious and oncologic complications [2]. Antiretroviral therapy (ART), which has been available for more than two decades, can strongly suppress HIV replication and reduce the risk of secondary diseases [2].

In 2021, more than 90,800 people in Germany were living with an HIV infection [3]. However, the risk of late diagnosis remains high, with 32.9% of all initial HIV diagnoses associated with advanced immunodeficiency, including clinical AIDS or a CD4 cell count <200 cells/µL, indicating severe immune system damage caused by HIV [3]. Persons known as late presenters (LPs) have a CD4 cell count of less than 350 cells/µL at initial medical presentation or already have an AIDS-defining disease [4]. The HIV pre-exposure prophylaxis (PrEP) has become an important strategy to prevent HIV infections in Germany [5]. Since 2019, every person with public health insurance has the right to comprehensive counseling, provision of medications, and beneficial examinations such as regular testing for sexually transmitted infections (STIs) [6]. Although ART has significantly improved patient outcomes, challenges in health services for HIV patients should not be underestimated. Language barriers and issues in providing continuity of care (CoC) are daily obstacles in offering guideline-oriented care.

In a rapid review from 2007 to 2017, the majority of studies showed proven or preliminary efficacy at improving targeted outcomes when applying eHealth measures for providing CoC [7]. It is not surprising that in the recent past, several mobile health applications (mHealth) have been developed to allow patient self-management or prevention, and improve medication and ART adherence in HIV therapy [8,9,10,11]. Studies at the international level have shown positive effects of mHealth interventions: for instance, cell phone use and text-messaging for communication between physicians and patients in the United States [8,12]. Sun et al. demonstrated in a meta-analysis that the use of bidirectional SMS in the care of HIV patients can have a positive effect on ART [13].

In Germany, only a few mHealth applications—specifically mobile apps for HIV/AIDS—are available. The app “Prepared”, for example, offers a simple PrEP medication reminder function [14]. “MyTherapyApp” includes a medication reminder, as well as a symptom diary, which can be printed out and thus presented to physicians [15]. Moreover, the app “Life4ME+” features appointment scheduling with a physician, display of test results, a prescription history and medication reminders [16].

However, these apps have been developed as standalone systems, lacking direct integration into physicians’ clinical workflows. Studies on other diseases have shown that direct integration of patient apps into clinical workflows, through remote consultations via video and chat, SMS, or email, can have a positive effect on patient care [17,18,19,20]. In addition, technical integration into the electronic health record (EHR) needs to be available in order to facilitate patient-centered care [21,22,23]. However, a further success factor for such apps is a high rate of usability and acceptance by the users [24,25,26,27]. A study by Raeesi et al. indicated that HIV/AIDS apps often do not meet user requirements [28].

To address these gaps, the Communication and Tracing App HIV (COMTRAC-HIV) project aims to develop and enable an integrated system for HIV-related care, specifically targeting LPs and PrEP users, through a mobile app. COMTRAC-HIV has been funded as a two-year project by the Hessian Ministry for Digital Strategy and Development. The goal is to develop an initial app prototype that allows communication between physicians and patients via video and chat and that offers a symptom diary to be filled in by the patient enabling the doctor to monitor the patient’s health status. We therefore conducted this study at an early stage of development to determine which aspects are relevant to the care of HIV patients and the use of the COMTRAC-HIV app from the point of view of HIV experts.

The objectives of the study were (1) to investigate the relevant organizational conditions for operating the COMTRAC-HIV app and (2) to determine the app functionalities necessary from a physician’s point of view for its integration into patient care.

## 2. Materials and Methods

### 2.1. Design

We followed a human-centered design (HCD) approach according to the “ISO 9241-210:2019 Ergonomics of human-system interaction-Part 210: Human-centered design for interactive systems”, with the goal of involving the stakeholders in the development process [29,30,31,32,33,34].

**Figure 1:**
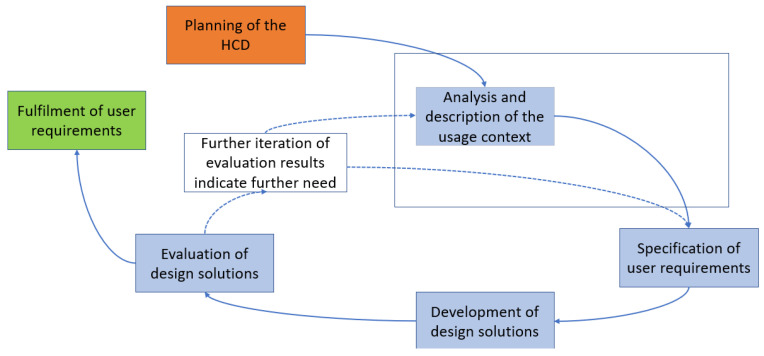
Human-centered design according to the “ISO 9241-210:2019 Ergonomics of human-system interaction–Part 210: Human centered design for interactive systems” [33,34].

The typical HCD process is shown in the figure below. The HCD is divided into five phases from “Planning of the HCD” to “Evaluation of design solutions”. The HCD is to be carried out iteratively until the requirements for a system are met from the users’ point of view. In this publication, we focus on the highlighted phase (see Figure 1) in addressing our research objectives, which will be explained in more detail in the following.

**Figure 2:**
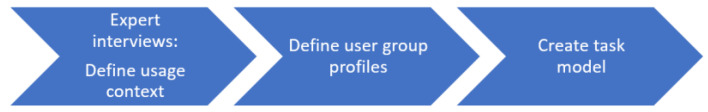
Steps performed in this study according to Geis and Polekehn [33].

We followed the methods of Geis and Polkehn, as shown in Figure 2, which provide guidance on how to perform the steps in the individual phases of ISO 9241-210:2019 [33,34]. We interviewed HIV experts to determine the usage context for the app to be developed. The usage context includes the users of the system with their characteristics, the tasks that the users want to perform with the system, the environment in which the system is to be used and the resources that are available to the users [33]. The expert interviews were conducted and reported following the Standards for Reporting Qualitative Research (COREQ) [35]. A checklist for COREQ items considered is provided in Supplementary File S1.

Based on the results of the expert interviews, user group profiles were defined according to “ISO/IEC 25063:2014 Systems and software engineering—Systems and software product Quality Requirements and Evaluation (SQuaRE)—Common Industry Format (CIF) for usability: Context of use description”, which specifies the content of detailed context of use descriptions for intended systems [36]. Each user group is therefore described with the following characteristics:

- Socio-demographic characteristics: Age, gender, and other socio-demographics.
- Task-related characteristics: Tasks, as well as knowledge, skills, resources, and motivation to perform tasks with the planned system.
- Organizational characteristics: Current organizational aspects, reliability, skills, and willingness for change.
- Psychological and social characteristics: Cognitive skills, cultural background, language, and literacy.
- Physical and sensory characteristics: Physical and sensory condition of the users.
- The extracted usage context information was applied to construct task models that the app should contain and that form the basis for the app functionalities. A task model includes both tasks and related subtasks. It is used to clarify which tasks are to be performed with the app and to understand which paths the user chooses when performing the task and allows for designing a system that is suitable for the application [33].

### 2.2. Setting and Sampling

For the expert interviews, we used a purposeful sampling in which experts in HIV were invited to participate in the study [37]. An expert was defined as a person who has comprehensive and authoritative knowledge in a particular domain gained through professional practice, training, and experience [38]. Inclusion criteria for study participants were that they work in an inpatient or outpatient setting anywhere in Germany with a focus of HIV/AIDS patient care, that they have completed their medical degree and that they have completed a specialist qualification in internal medicine, which means a minimum experience of six years in medicine. Based on these criteria, eight potential study participants, known by the authors, were invited in accordance with sample size recommendations for requirement and usability studies [39,40]. The participants were informed that the study was to be conducted in the context of the COMTRAC-HIV project.

The participants were recruited by email. The invitation including a study information letter which described the purpose of the study was sent in August 2022. If no reply to the email was received within two weeks, the experts were then contacted by telephone. Recruitment of participants continued until data saturation was reached so that all deductive categories (see Section 2.3) were sufficiently represented.

### 2.3. Data Collection

An interview guide was created in German within a disciplinary team of computer scientists, physicians, psychologists, and epidemiologists including the HIV-Center Frankfurt. For purposes of this publication, we translated the guide into English (see Supplementary File S2). We developed the interview guide according to the approach suggested by Hellferich and Kuckartz [41,42]. Therefore, we defined key terms (shown in Table 1) related to the research objectives, before defining deductive categories. Deductive categories serve as theory-based structuring dimensions for qualitative content analysis [43]. All authors jointly defined the key terms.

Based on these key terms, we defined the following deductive categories:

- Current care workflow: Organization of patient care of LPs and PrEP users and current form of communication with the patients.
- App functionalities and possible benefits for LPs and PrEP users.
- Further app functionalities: Additional information or medical findings whose entry in the app could have special significance for physicians or patients.
- Problems/disadvantages: Any possible negative impact of the planned app on patient care.
- Benefits for HIV experts: Possible incentive that the app offers for the physicians providing care.
- Possible adaptation of the clinical workflow: Adaptation of typical clinical processes in everyday medical practice using the app.

In addition, study participants were asked in the interviews about their gender, experience in treating HIV patients, their medical specialization, how often they treat LPs and PrEP users in their daily work and which languages non-German-speaking LPs and PrEP users speak.

Before the interviews were conducted, an internal pretest was performed together with the HIV-Center Frankfurt. The pretest indicated that only minimal changes in the order of the questions of the interview-guide were necessary.

Participants were interviewed online using video-conferencing software, between September and December 2022. The interviews were conducted in German by two researchers: RN, with an informatics background and SK, with an epidemiological background. Both have experience in qualitative research. Each researcher interviewed four participants. As mentioned above, a study information letter informed participants of the study’s aims and that it was part of the COMTRAC-HIV project. However, no relationship was established with the participants prior to study commencement.

After signing the study consent form to participate in the study, the recording started and the first question was asked. In order to avoid interruptions during the study no further persons were present. Interviews were conducted once and not repeated. They ranged from 32 to 66 min in length.

### 2.4. Data Analysis and Processing

The audio recording of the interviews was transcribed in a paraphrased form using Microsoft Word. The paraphrase includes formulation of a text passage in the researcher’s own words [44].

RN, SK and AV paraphrased the interviews, while they were cross checked by each other, by listening to the interview again and making any corrections or additions if required. To ensure data protection, the recorded audio files were uploaded to a secure server of the authors. Only the interviewers had access and were responsible for data management. After the paraphrases were finished, the recording files were deleted from the server. All identifying data in the paraphrases were removed to ensure anonymization.

For data analysis, each paraphrased transcription was assigned to one of the six categories mentioned in Section 2.3. However, no further changes were made to the category system during the analysis. All authors discussed any paraphrased text that could not be directly assigned to a category. To synthesize the results of the interviews, paraphrased texts from the experts were used which best represented the content of a category. JS translated the paraphrased texts from German to English; AM and RN cross-checked them. The paraphrased texts were used to document the user groups (LPs and PrEP users) in a table format. For this purpose, the information extracted from the paraphrasing was arranged according to the respective user group and matching the user characteristics of the ISO/IEC 25063:2014 [36]. Further sub-characteristics were also created, which were discussed with all authors. For example, the socio-demographic characteristics were supplemented by additional sub-characteristics such as “distance to treatment center/HIV clinic” or “insurance status”. The task model was then defined and visually represented in a figure listing tasks and subtasks, and approved by all authors [33].

## 3. Results

### 3.1. Interviews

#### 3.1.1. Participants

All eight experts contacted accepted the study invitation and subsequently participated in the study. The study participants’ characteristics gathered during the interviews are listed in Table 2.

#### 3.1.2. Main Themes by Category

In the following, we present the results of the interviews; they are organized by the categories defined in Section 2.3. References for selected paraphrased texts are given for each statement, abbreviated by “P” and numbered in ascending order (e.g., P1). All paraphrased texts are included in Supplementary File S3.

##### Current Care Workflow

When the expert participants were asked to describe the current care workflow of HIV patients, they stated that LPs are often diagnosed in a hospital. Many of the patients have previously had various indicator diseases and infections, but were not previously diagnosed as having HIV:

*LPs are often individuals who go from one doctor to another for months/years without an HIV diagnosis and have repeated HIV indicator diseases that are not detected, such as recurrent herpes zoster, recurrent infections, B-symptomatology, and lymph node swelling, and who are not offered HIV diagnostics*.

(P1)

After their HIV diagnosis, these patients are subsequently treated in an HIV outpatient clinic in most cases (P2).

With PrEP, the initial presentation of a patient takes place in the context of a consultation, during which appropriate examinations (e.g., by taking blood samples) are performed. Regular appointments are offered to PrEP users within the clinic outpatient departments. In addition, there are emergency appointments for patients suffering acute infections such as sexually transmitted infections (STIs) (P3–P5).

The HIV experts also indicated that there are no differences in treatment between the individual groups of patients (P6–P7). However, pregnant women were named as exceptions in this context, as more intensive care must be provided before and after they give birth (P6–P7). In addition, as patients without German language skills often face language barriers, appointments are arranged together with a translator (P6, P8).

When asked which communication tools they already use to communicate with patients, all experts stated that they primarily use email and telephone (P9–P10). Only two experts already use telemedical applications that, for instance, allow video calls. They mentioned problems in the use of and legal requirements for these applications (P11–P12). Furthermore, one study participant expressed concerns about using their private smartphone for communication with patients:

*Some patients have the participant’s phone number and send photos of problems without restraint—bad in terms of privacy*.

(P13)

##### App Functionalities and Possible Benefits for PrEP Users and LPs

The experts indicated that the advantages of a symptom diary are that it can identify small changes in the health status of LPs over time and that it is possible to derive trends based on this (P14–P15). However, concerns were also raised that some LPs may not be able to record symptoms. The symptom diary can be used by well-adjusted and engaged LPs who have been in treatment for some time and in cases when patients and physicians know each other (P16–P17):

*A telemedical app is conceivable for well-established/admitted HIV patients with a viral load below the detection limit. However, this requires good mutual knowledge and enables communication based on the available information.*

(P17)

When the experts were asked which symptoms, intensities and critical constellations should be recorded in the symptom diary to identify any side effects of medication or an HIV infection in PrEP users, different aspects were mentioned (P18):

*For PrEP users who are afraid of contracting HIV disease or other diseases. And because there are often side effects of the prescribed pills at the beginning of therapy, but they stop after a few months*.

(P18)

The experts indicated that symptom intensity would be less relevant for PrEP users because symptoms are not severe and therefore intensity is not critical (P19–P21). Critical symptom constellations for PrEP included a variety of symptoms related to new HIV infection and possible STIs. (P22–P24):

*For new HIV disease, fever, lymph node swelling, general feeling of illness would be characteristic*.

(P22)

On the other hand, the following symptom-tracking information was noted for LPs:

*Alarm symptoms: Fever, rash, visual disturbances, shortness of breath, chest pain, bleeding signs, opportunistic, and non-opportunistic*.

(P25)

It was indicated that for LPs the intensity as well as frequency and duration should be recorded on a scale (P26). LPs are reported to have a wide variety of critical symptom constellations, often related to health status or complications (P27–P30).

In addition to somatic complaints, psychological problems were addressed in LPs and PrEP users (P31–P32):

*Classic symptoms that can mask depression include: loss of libido, loss of drive, sleep disturbance, headaches, and the whole psychosomatic range that indicates depression/anxiety*.

(P31)

With regard to the app’s chat functionality, participants emphasized that it could provide a quick answer to acute questions of LPs (within a few hours) and, if necessary, could avoid a visit to the emergency room or an appointment during consultation hours (P33). One expert also pointed out that it would be useful if the findings (e.g., blood results), which are collected every 3 months for each PrEP user, could be transmitted directly to the patient via the chat, as their transmission is currently very time-consuming for the experts. Open questions about the findings could then also be clarified via chat or video call (P34).

##### Further App Functionalities

The experts stated with regard to other functionalities or information they considered, the app should display CD4 results to LPs. This could be helpful for patients during the course of the disease, to increase their interest in the disease and help them to feel more secure through a stable course of the disease (P35). Participants furthermore expressed that the app should offer a means to upload information and findings from patients as well as physicians who do not work in an HIV outpatient clinic but are involved in the health care of patients (P36–P37).

In addition, the documentation of medication intake was mentioned, especially with respect to PrEP users. Here, the experts stated that with daily documentation, PrEP medication compliance can be determined and identified when a PrEP medication is stopped (P38–P39):

*For PrEP, as needed and daily: document and to determine compliance, i.e., review/show tablet use, as this is where errors often occur […].*

(P38)

In addition, the app should also remind users of appointments for checkups (P38).

##### Problems/Disadvantages

The experts were also asked whether the use of the app could also have a negative impact on patient care. It was noted that the app should be easy to use and only be used when necessary, and that important markers should be entered (P40). Furthermore, the app should not replace the patients’ regular quarterly visits (P41). The participants were also critical of the possibility that information could be misunderstood or misinterpreted by patients, arouse their fears or not be noticed (direct feedback lacking in contrast to the telephone call). Therefore, the app is not suitable at the beginning of treatment (the sensitive phase in which direct contact is very important), but rather for those patients who have “arrived” with their diagnosis, to request information in between or to clarify questions quickly (P42). In this context, the study participants also mentioned that there were certain patient groups that could handle the app particularly well or particularly poorly:

*The age group of HIV patients is able to use a smartphone (tends to be younger), no restrictions for specific subgroups […]*

(P43)

*PrEP users: especially good, mostly younger, tech-savvy—mostly (high) school graduates, grasp it better.*

(P44)

The language problem, especially with LPs, was also mentioned (P45–P46):

*A multilingual app is very important; the app should include a translation feature that facilitates communication and can translate what the patient is talking about live and play back the translation on the screen.*

(P46)

##### Benefits for HIV Experts

The experts were also asked what advantages the app offers HIV experts and in what way the app can support their work. Among other things, the experts mentioned that more patients could be cared for (P47). In addition, the app would facilitate communication with patients who are unable to come to consultation hours for various reasons (P48–P49):

*Ability to communicate with patients when they are not on site and discuss how the year was progressing for them. This would have helped a lot during the COVID period, as some patients came out of fear.*

(P48)

The experts also indicated that processes could be simplified, such as patients being able to access findings directly and order prescriptions via an app. In addition, unnecessary visits to the outpatient clinic could be avoided, as findings could be discussed directly via video call and prescriptions for medication directly ordered (P50–P51).

##### Possible Adaptation of the Clinical Workflow

Towards the end of the interview, the HIV experts were asked to what extent the clinical processes in their daily work would have to be adapted if the app were used in patient care. Above all, the experts pointed out the need to keep additional time slots free for use of the app (P52–P54):

*Free up time to maintain the app—1–2 h/week.*

(P52)

Furthermore, all study participants indicated that they would like to access the app via their work computer. No messages should be forwarded to their private or work smartphone (P55–P56). To enable connectivity in the physicians’ clinic, the study found that an interface to the corresponding clinic software would need to be available (P57).

### 3.2. User Group Profiles

Based on the results of the interviews, the corresponding user group profiles for LPs and PrEP users were described using the user characteristics of ISO/IEC 25063:2014 [36]. In the following, we provide a short overview of the user group profiles; the full description is available in Supplementary File S4.

#### 3.2.1. Late Presenters

Socio-demographic characteristics highlight that male patients are typically diagnosed in their late 30 s, while women are usually post-menopausal. Migrants are more likely to undergo screening, indicating higher risks or challenges related to HIV. The residential environment of HIV-positive individuals can vary between urban and rural areas, and many face psychosocial and economic difficulties. Lack of awareness about the disease is also common, and some patients have to travel long distances for specialized treatment.

Task-related characteristics show that people from different groups, including young, old, homeless, and migrants, have experience using mobile phones. However, elderly individuals may not have a mobile phone or be familiar with its use. Having an HIV app on a mobile phone is considered important, but some individuals may be unable to use it due to their medical condition. The app should provide symptom-tracking, facilitate contact with health-care providers, and promote communication and interaction between individuals and their providers.

Psychological and social characteristics highlight the distress, anxiety, and uncertainty experienced upon receiving an HIV diagnosis. Individuals often rely on the app or phone to communicate with health-care providers, especially for anxiety-related issues. Concerns about data privacy have been expressed. Multilingual support is important to accommodate individuals with diverse language backgrounds.

Organizational characteristics emphasize the role of primary care physicians in HIV care, including diagnosis and regular check-ups. Adherence to appointments is crucial, so the app should provide appointment reminders to support therapy management.

Physical and sensory characteristics include the CD4 count, comorbidities, psychiatric conditions, and medication interactions/side effects. The app should assess symptom intensity using scales, with immediate attention required for specific combinations of symptoms.

#### 3.2.2. PrEP Users

Socio-demographic characteristics indicate that the majority of PrEP users are young, male, and well-educated. However, eligible women often go unidentified or are unaware of PrEP.

Task-related characteristics show that PrEP users are technologically savvy and comfortable with mobile devices and apps. They have knowledge about HIV therapy and STIs. The COMTRAC-HIV app should enable users to schedule appointments for symptoms, ask spontaneous questions, handle follow-ups, identify acute treatment needs, record symptoms, rate symptom intensity, pre-order prescriptions, and access findings. Technological devices such as mobile devices and fever thermometers are also useful resources for PrEP users.

Psychological and social characteristics indicate that PrEP users are comfortable with mobile devices and have knowledge about HIV and STIs. However, they have concerns about data privacy. Providing the app in multiple languages would be helpful for non-German-speaking users.

Organizational characteristics suggest that some PrEP users require referral from primary physicians, while others do not. In some cases, the primary physician prescribes the medication. PrEP users in the study demonstrate reliability and compliance by taking their medication regularly, undergoing STI screenings, and having regular follow-up appointments.

Physical and sensory characteristics reveal that some users may have pre-existing psychiatric conditions and may experience acute health changes and symptoms related to STIs and side effects from PrEP medications. Fever, signs of inflammation, or pain during sexual activity are examples of critical symptom constellations.

### 3.3. Tasks

**Figure 3:**
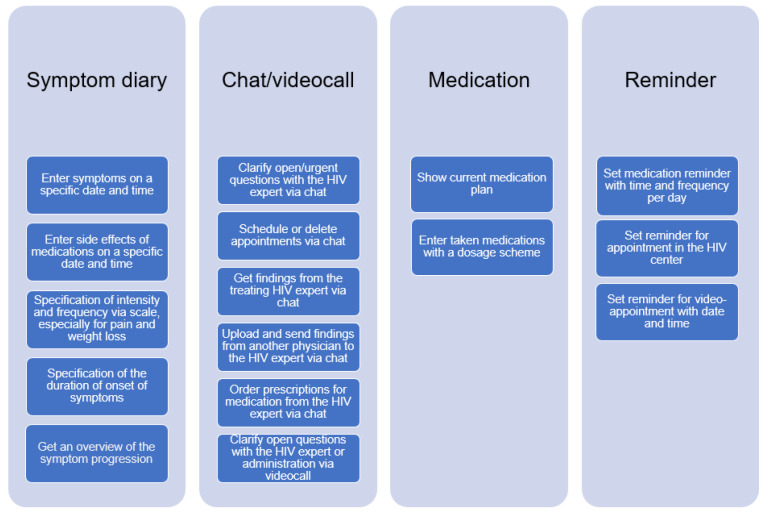
Overview of tasks for the COMTRAC-HIV app.

In this section, we describe the tasks derived from the user group profiles. Overall, the following tasks were identified: symptom diary, chat/video call, medication overview, and reminder. Each of these tasks has sub-tasks, which are shown in Figure 3 below.

## 4. Discussion

This qualitative study is a first step towards development of a mobile app enabling integrative care of HIV-infected individuals in Germany. In concrete terms, the study investigated the relevant organizational conditions for the COMTRAC-HIV app and functionalities necessary to integrate the app into patient care. These two research objectives are discussed in more detail in the following sections.

### 4.1. Discussion of Results

#### 4.1.1. Organizational Conditions

The results of the interviews show differences between LPs and PrEP users regarding possible app usage. The experts stated that LPs are often non-native German speakers and thus many of them have language problems. Therefore, the app must consider a wide variety of languages, especially in a country of immigration such as Germany [45]. However, the app “Prepared” mentioned in the introduction is only available in German, whereas “Life4ME+” (https://www.prepared-app.de) and “MyTherapy” (https://www.mytherapyapp.com/de/diagnose-hiv-wie-eine-apphilft-mit-der-immunschwaeche-umzugehen) offer different language versions [14,15,16]. To cover as many languages as possible, one expert suggested an automatic translation function. In this context, it must be determined whether a corresponding implementation effort justifies a possible benefit of this feature. Panayitou et al. evaluated in a study 15 different apps which provide an automatic translation function. The authors concluded that all apps require caution and consideration when used in healthcare, therefore professional translators should not be replaced [46]. Whether such a function is practicable for the COMTRAC-HIV app, can only be tested in further studies.

It was also stated in the interviews that LPs should not use the app at the beginning of their diagnosis because critical information could be misunderstood. Some LPs are often diagnosed at an HIV center, while PrEP users have a self-interest in protecting themselves from HIV infection. Some LP individuals may be unable to use an app due to their medical condition. Therefore, the experts suggested that only well-adjusted and engaged LPs should use the app. However, initial studies show that the number of LPs in Germany may have increased during the COVID pandemic [47,48]. Physicians should distinguish in each case whether an app can be useful or not.

On the other hand, the experts consider PrEP users to be more apt at using the app—they are usually younger and more technology-oriented. The experts’ statements are supported by other studies that have found that acceptance of mHealth apps among PrEP users is generally high [49,50]. Hence, we will examine in further studies on whether usage and acceptance differ between the two user groups. However, concerns about data privacy, e.g., when the app emits warning messages, constitute a characteristic both groups have in common. This is similar to other studies which have already shown that people living with HIV have a high level of awareness regarding the privacy and security of their health data [51,52]. The design and implementation of the app must ensure that patients using the app in their daily lives cannot be identified by other people as HIV-infected individuals or PrEP users.

When the experts were asked what advantages the COMTRAC-HIV app would offer, they indicated process improvements such as treating more patients and avoiding unnecessary visits. However, one of the things the experts said they would need to do was free up a few hours a week in their schedules to use the app. In the literature, one study evaluated telephone reminders for appointments and measured a decrease in missed appointments from 11.4% to 7.8% [53]. Dillingham et al. demonstrated in a 12-month study with an mHealth intervention that retention in care increased from 51% to 88% [54]. Once the app has been developed, we plan to investigate, in addition to acceptance and usage, corresponding process improvements in the care of LP and PrEP users.

#### 4.1.2. App Functionalities

The app functionalities can contribute effective management of HIV-related information and resources (e.g., prescriptions and findings), promote communication and information-sharing between patients and physicians (via video and chat), schedule appointments, and foster patient autonomy and self-management skills (e.g., via the symptom diary with symptom history, medication reminders). However, we need to clarify in the next development steps how the system can be technically linked to the EHR of physicians. Several studies have been performed in the past using standards like Health Level 7 Fast Health Interoperability Resources (HL7-FHIR) to exchange data with local EHR [21,55]. Moreover, as part of the creation of the design solutions—one of the next steps in ISO 9241-210, the functionalities must be converted into a user-friendly interface featuring intuitive navigation [33,34]. We are planning to organize focus groups with people affected by HIV to include in our discussion on initial app design and to test the usability of the design. The design can be tested, for instance, using a clickable prototype and with the method of “Thinking Aloud”, where participants are to solve tasks with the prototype while sharing out loud what they think about the app [56].

The proposed app does, in fact, have similar functionalities to other available apps in German-speaking countries, such as “MyTherapy” and “Life4ME+”. However, the COMTRAC-HIV app will not address every HIV-infected individual, but specific LPs and PrEP users; it will also collect data on their health status via the symptom diary, as well as enable video and chat communication with the treating physician. Functionality, acceptance, and usability are nonetheless insufficient if the treating physicians do not use the app. Physicians only offer health services if they are also remunerated for their efforts [57]. Therefore, the COMTRAC-HIV app is to be certified as a digital health application (*Digitale Gesundheitsanwendungen* or DiGA) in Germany, which would mean it could be prescribed by physicians [58]. Since 2019, physicians can prescribe DiGA if they have been approved by the Federal Agency for Drugs and Medical Devices (BfArM) in a testing procedure. The approval test examines aspects such as the positive effect on patient care as well as data protection and usability. To our knowledge, there is no DiGA for the prophylaxis and therapy of HIV in Germany.

### 4.2. Discussion of Methods

For this study, we chose expert interviews for gathering extensive first-hand information and insights. When analyzing expert opinions, it must be borne in mind that personal interests or conflicts of interest cannot be completely ruled out. Especially as Germany is the first country in the world to offer patients the option of reimbursement for mHealth apps through statutory health insurance [59] and this is a completely new situation, the impact on physicians’ attitude towards mobile health applications is yet unknown.

With regard to the recruitment strategy, we opted for purposive sampling, where participants, known by the authors, were invited. Therefore, it cannot be excluded that a selection bias or a response behavior in terms of “(social) desirability” is present.

This affects the generalizability of the study because generalization is only possible to the population from which the sample was drawn [60]. Furthermore, this form of recruitment resulted in a possible gender bias, as seven of the eight participants were male. However, related research indicates that gender-based differences can occur, e.g., in later usability of the app, whereas other studies indicated no difference [61,62,63].

Our study is limited to eight participants. However, as with any qualitative research, results are not generalizable or representative [64]. We made a conscious decision on the number of participants, as we found a corresponding saturation of information in data analysis [65]. This is reinforced by a systematic review of Hennink et al. which states that qualitative studies can reach saturation at relatively small sample sizes [66]. Furthermore, we identified other studies that used small sample size for gathering initial requirements for development of an app [39,40,67]. Although the sample was small, the study represented a group of HIV experts with a wide range of experience. Expert interviews are a suitable instrument for eliciting requirements at an early stage of development, as numerous other studies have shown in the past [68,69,70]. This method allows an intensive exchange with a small group of participants and produces more convincing data than quantitative studies do [71,72].

Furthermore, we consciously decided not to include patients in the initial interviews, but to only seek expert opinions in our efforts to investigate the organizational conditions and to determine which functionalities for the app are needed to support HIV care. As mentioned in Section 4.1, we plan to involve patients in the specific design solutions and further evaluation of the app.

In addition, the reliability and validity of the results of the present study must be discussed. Although the study was conducted by an interdisciplinary team including experts from the HIV Center Frankfurt, potential bias cannot be excluded, as the final transcripts as well as the results of the study were not returned to the participants for discussion or correction.

According to the definition of user group profiles and tasks, this methodology is limited to the suggestion of Geis and Polekehn et al. [33]. However, this method is recommended when following an HCD according to the ISO 9241-210 [34] and is used in a certification program called “UXQB^®^ Certified Professional for Usability and User Experience—Advanced Level User Requirements Engineering” [73].

## 5. Conclusions

This qualitative study involved experts of HIV to investigate the relevant organizational conditions and functionalities from the physician’s point of view for operating the COMTRAC-HIV app in patient care.

The results indicate different functionalities like a symptom diary, chat/video functionality, appointment organization, sharing findings, medication overview, and reminder for medications and appointments. The study also highlights differences in the care of PrEP users and LPs, e.g., language barriers and app privacy and security, which should be addressed in the further development of the app. However, further work and studies are necessary, such as the assessment of the app’s sustainability, integration into existing healthcare systems and long-term effects on patient outcomes.

## Figures and Tables

**Table 1:** Key terms as a basis for the interviews.

Research Objective	Key Terms
Investigate the relevant organizational conditions for operation of the app	Current workflow of LP patient care
	Current workflow of PrEP patient care
	Differences in PrEP users and LP patient care
	Derivations from the current clinical workflow
	Participation of different specialists in patient care
	Involvement of other institutions
	Communication with patients
	Possible benefits for physicians
	Adaptation of the current clinical workflow
Necessary functionalities for the app from the physician’s point of view in order to integrate the app into patient care	Planned app functionalities: Symptom diary, chat, and video
	Relevant further app functionalities

**Table 2:** Characteristics of study participants.

Characteristics	Specifics	No. of Participants to Which the Characteristic Applies (n = 8)
Gender	Male	7
	Female	1
Experience with HIV patients	0–5 years	1
	6–15 years	1
	16–25 years	3
	≥26 years	3
Medical specialization	Internal medicine	3
	Internal medicine/further training in infectiology	4
	Internal medicine/further training in nephrology	1
Frequency of care of LPs	Several times a week	5
	Monthly	3
Frequency of care of PrEP users	Several times a week	6
	Monthly	2
Non-German languages of PrEP users and LPs	Arabic	1
	Turkish	2
	Russian	2
	English	2
	Romanian	1
	Spanish	1
	Italian	1
	Languages spoken in sub-Saharan Africa (e.g., Kenya, Ghana)	1

## Data Availability

The data presented in this study are available on request from the corresponding author. The data are not publicly available due to privacy restrictions.

## Supplementary Materials

The following supporting information can be downloaded at: https://www.mdpi.com/article/10.3390/healthcare11152180/s1, Supplementary File S1, Supplementary File S2, Supplementary File S3 and Supplementary File S4.
