# Supplementary material for: Interviews with HIV Experts for Development of a Mobile Health Application in HIV Care—A Qualitative Study
Source: Healthcare (Basel). 2023 Aug 1;11(15):2180. doi: 10.3390/healthcare11152180 (PMC10418895; doi:10.3390/healthcare11152180)
Supplement: Supplementary file 1 [file healthcare-11-02180-s001.zip › Supp Files/Supplementary File S2.pdf]

COMTRAC HIV Interview Guide for Expert Interviews

PrEP: Users of HIV pre-exposure prophylaxis; LP: Late Presenter

**Introductory question**	**Sub-questions**	**Topic and flow of conversation**
• Greeting, state name and position at the institute<br>• Thank you very much for taking the time for this interview.<br>• As you know, the COMTRAC-HIV project aims to enable telemedical monitoring for LP and PrEP users, which includes a symptom diary and a communication option between patients and treatment providers.<br>• We would now like to talk to you about how outpatient care currently looks for these two groups. What is going well, what is perhaps going less well, and what contribution could an app make to overcoming weaknesses?<br>• Our conversation will last about 45-60 minutes.<br>• Do you have a question in advance?<br>• I would like to record the interview, but it will be evaluated pseudonymously so that no conclusions can be drawn about your person later. Do you agree to this?<br><br>*If yes, start recording. If no, take notes.*		Welcome, explanation of the project and the framework conditions for the interview
First of all, imagine a typical patient from the LP group. I would like to ask you to describe to me from your practical experience how their outpatient care usually works. We are particularly interested in how the patients come to you.	• Who issues referral slips? Is it possible to see a specialist directly without a referral from the family doctor?<br>• Are other institutions (such as AIDS support) involved?	Actual state of care, contact and cooperation at LP
Now imagine a typical PrEP user. I would now like to ask you to describe to me from your practical experience how their outpatient care usually works. We are particularly interested in how the patients come to you.	• Who issues referral slips? Is it possible to see a specialist directly without a referral from the family doctor?<br>• Are other institutions (such as AIDS support) involved?	Actual state of care, contact and cooperation among PrEP users
Are there persons or groups of persons for whom you deviate from the usual care? If so, who are they, what do you do differently and what are the reasons for this?	• Which persons, groups of persons are they?<br>• What are the reasons for this?	Actual state: deviations in care, communication
Do you currently use other means of communication besides face-to-face appointments, such as email, telephone or telemedicine?	• If yes, which ones and for what?<br>• If not, why not?	

We have now talked extensively about how the care of HIV patients is currently organised in your clinic. Now we want to know how you think this care situation would change if telemedical monitoring were offered.<br>For information: Telemonitoring is the monitoring and evaluation of important health values from a distance.		Introduction Target state
If you imagine an app with a patient-managed symptom diary and communication options such as on-demand chat or video telephony, could these tools serve LP?	What could be achieved through ....?<br>• Symptom diary<br>• On-demand chat<br>• Video telephony	Target state LP: App functions and their benefits for LP
One more question about the symptom diary for LP: Are there symptoms that absolutely have to be asked there in order to recognize a critical state of the disease, side effects of medication or, for example, emerging sexually transmitted diseases in time?	• Should the symptom intensity be indicated?<br>• Are there certain constellations that you consider particularly critical?<br>• Should psychological problems be recorded in addition to somatic problems?	
In your opinion, could symptom diaries and on-demand chat or video telephony help PrEP users?	What could be achieved through ....?<br>• a symptom diary<br>• an on-demand chat<br>• Video telephony	Target state PrEP: App functions and their benefits for PrEP
Again, the question about the symptom diary for PrEP users: Are there any symptoms that absolutely have to be asked there in order to recognize an HIV infection, side effects of the medication or, for example, emerging sexually transmitted diseases in time?	• Should the symptom intensity be indicated?<br>• Are there certain constellations that you consider particularly critical?<br>• Should psychological problems be recorded in addition to somatic problems?	
Is there any additional information or findings whose entry in the app could have special significance for patients or practitioners?	• Significance for patients: Differences PrEP users vs. LP<br>• Importance for the practitioners<br>• Differences PrEP users vs. LP	Additional app function

Could the app perhaps also influence care negatively? Where do you see risks?	• Differences PrEP users vs. LP?	Problem/ Disadvantages
In your opinion, what incentive does the app offer for the doctors providing care?	• What added value do you personally see in such an app?<br>• At which points in the care process can the app support?	Functionalities from a doctor's perspective
Now please think about your daily work and typical clinical processes. If you were to use the app in patient care, i.e. view symptoms and receive chat and video requests, what clinical processes might need to be adapted?	• Differences PrEP users vs. LP?	
If you want to access the patient data: Through which end devices do you prefer to access it? (Mobile devices such as smartphone or tablet, computer etc.)	• What would be your personal expectations?	
Is there anything else we haven't talked about yet that could be relevant to the topic?		Closing
Finally, I have a few questions about you and the HIV patients you care for.	• How long have you had experience in caring for HIV patients?<br>• How often do you care for LP?<br>• How often do you care for PrEP users?<br>• Which languages do non-German speaking PrEP users and LP patients speak?	Selected information on the practitioner and patients
• Thank you very much for taking the time for this interview and giving us this insight into your work.<br>• May we contact you again if we have further questions during the course of the research project?<br>• Adoption		**End of the interview**
