# Supplementary material for: Interviews with HIV Experts for Development of a Mobile Health Application in HIV Care—A Qualitative Study
Source: Healthcare (Basel). 2023 Aug 1;11(15):2180. doi: 10.3390/healthcare11152180 (PMC10418895; doi:10.3390/healthcare11152180)
Supplement: Supplementary file 1 [file healthcare-11-02180-s001.zip › Supp Files/Supplementary File S3.pdf]

Example paraphrases from the interviews

Number	Paraphrase (translated into English)	Paraphrase (original German)	Expert Number
1	LPs are often individuals who go from one doctor to another for months/years without an HIV diagnosis and have repeated HIV indicator diseases that are not detected, such as recurrent herpes zoster, recurrent infections, B-symptomatology, lymph node swelling, etc., and who are not offered HIV diagnostics.	LP oft Personen, die über Monate/Jahre ohne HIV-Diagnose von einem Arzt zum nächsten laufen, wiederholt HIV-Indikatorerkrankungen haben, die nicht erkannt werden, wie z. B. rezidivierenden Herpes zoster, immer wieder auftretende Infekte, B-Symptomatik, Lymphknotenschwellung usw. und denen keine HIV-Diagnostik angeboten wird.	E4
2	LP are diagnosed as inpatient, treated until ready for discharge. Patient receives outpatient medications that were determined inpatient.	LP wird stationär diagnostiziert, behandelt bis entlassungsfähig. Patient:in bekommt ambulant Medikamente, die stationär festgelegt wurden.	E3
3	Consultation, initiation of PrEP medication, regular appointments, in between emergency appointments if infections to be treated occur.	Beratungsgespräch, Einleitung der PrEP Medikation, regelmäßige Termine, dazwischen Notfalltermine, wenn zu behandelnde Infektionen auftreten.	E6
4	PrEP users are not ill people (more clients than patients), if sick then mostly acute problems like STIs.	PrEP Nutzer sind keine kranken Menschen (mehr Klienten als Patienten), wenn krank dann meist akute Probleme wie STIs.	E4
5	For PrEP, a standard procedure is offered in the clinic outpatient department: classic consultation hours and appointments.	Für PrEP wird in Klinikambulanz Standardprogramm angeboten: klassische Sprechstunde mit Sprechstundenzeiten und Terminvergabe.	E2
6	Medical treatment differs with only pregnant women, contacts are also more frequent. Closer contacts sometimes with persons with poor knowledge of German, possibly with the involvement of social workers.	Die medizinische Behandlung weicht bei nur bei Schwangeren ab; auch die Kontakte sind häufiger. Engere Kontakte manchmal bei Personen mit schlechten Deutschkenntnissen,	E1

Number	Paraphrase (translated into English)	Paraphrase (original German)	Expert Number
	The medical treatment does not deviate otherwise.	eventuell unter Einbezug von Sozialarbeitern. Die medizinische Behandlung weicht sonst in der Regel nicht ab.	
7	Overall, rather not. Pregnant women require close supervision and cooperation before and after birth.	Insgesamt eher nicht. Bei schwangeren Frauen ist engmaschige Betreuung und Kooperation vor und nach der Geburt nötig.	E5
8	Problem of language barriers - large number of Ukrainian patients. Appointments are arranged so that a familiar translator is present. Otherwise electronically, by mobile phone via Google Translate. Patients cared for.	Problematik Sprachbarrieren - Vielzahl ukrainischer Patienten. Termine werden so gestaltet, dass vertrauter Übersetzer mit dabei ist. Ansonsten elektronisch, per Handy via Google Translate. Betreute Patienten.	E3
9	A lot of work has to be done via email and it is good when patients use email as well, as email can be easily incorporated into the busy daily routine between patient appointments. It's difficult to find time for phone calls.	Es muss viel per E-Mail abgearbeitet werden und es ist gut wenn Patienten ebenfalls E-Mails nutzen, E-Mails gut einbaubar in den hektischen Alltag zwischen Patiententerminen, schwierig Zeit für Telefonate zu finden.	E6
10	Email is used, but difficult since very many emails per day. Time is very tightly scheduled with patients. Only time for e-mails in the evening, often from home.	E-Mail wird genutzt, aber schwierig da sehr viele E-Mails pro Tag. Die Zeit ist sehr eng getaktet mit den Patienten/Patientinnen. Erst abends Zeit für E-Mails, oft von zuhause.	E3
11	Not many patients use this - requires technical experience.	Nicht viele Patienten nutzen telemedizinischen Applikationen - erfordert technische Erfahrung.	E4
12	The problem with telemedicine is that only tools that are compliant with legal requirements can be used, others are not billable.	Problem bei Telemedizin ist, dass nur Tools genutzt werden können, die konform mit den rechtlichen Anforderungen sind, andere sind nicht abrechnungsfähig.	E5

Number	Paraphrase (translated into English)	Paraphrase (original German)	Expert Number
13	Some patients have the participant's phone number and send photos of problems without restraint - bad in terms of privacy.	Manche Patienten haben die Handynummer des Befragten und schicken hemmungslos Fotos von Problemen - schlecht für die Privatsphäre.	E3
14	Advantages of the symptom diary for practitioners: Documentation of even small changes over time. Derivation of trends possible.	Vorteile des Symptomtagebuchs für Behandler: Dokumentation auch kleiner Veränderungen im Zeitablauf. Ableitung von Trends möglich.	E2
15	The symptom diary could lead to a better assessment of the course of e.g. fever (when the practitioner fears that a patient is getting worse).	Das Symptomtagebuch könnte zur besseren Einschätzung des Verlaufs z. B. bei Fieber führen (wenn der Behandler befürchtet, dass es einem Patienten schlechter geht).	E1
16	It is unrealistic for patients to document their findings themselves.	Es ist unrealistisch, dass Patienten/Patientinnen ihre Befunde selbst dokumentieren.	E4
17	A telemedical app is conceivable for well-established/admitted HIV patients with a viral load below the detection limit. However, this requires good mutual knowledge and enables communication based on the available information.	Eine telemedizinische App bei gut etablierten/eingestellten HIV-Patienten/-Patientinnen - Viruslast unter Nachweisgrenze – vorstellbar. Das setzt jedoch gutes gegenseitiges Kennen voraus und ermöglicht Kommunikation auf Basis der vorhandenen Informationen.	E3
18	For PrEP users who are afraid of contracting HIV disease or other diseases. And because there are often side effects of the prescribed pills at the beginning of therapy, but they stop after a few months.	Bei PrEP, die Angst haben wegen einer HIV-Erkrankung oder anderen Erkrankungen. Und weil es zu Therapiebeginn häufig Nebenwirkungen der verschriebenen Tabletten gibt, die aber nach wenigen Monaten aufhören.	E1
19	Perhaps a scale for pain and other symptoms.	Vielleicht eine Skala für Schmerzen und andere Beschwerden.	E6

Number	Paraphrase (translated into English)	Paraphrase (original German)	Expert Number
20	Symptom intensity is less important for PrEP users, as symptoms are usually not severe, only symptom - yes/no.	Symptomintensität bei PrEP weniger wichtig, da Symptome normalerweise nicht schwer, nur Symptom ja/nein.	E5
21	Can be, but does not have to be, since intensity is not so crucial, but rather the detection of a disease, since this must be treated for PrEP to work.	Kann, muss aber nicht, da die Intensität nicht so entscheidend ist, sondern die Erfassung einer Krankheit, da diese behandelt werden muss, damit die PrEP funktioniert.	E3
22	For new HIV disease, fever, lymph node swelling, general feeling of illness would be characteristic.	Für neue HIV-Erkrankungen wäre Fieber, Lymphknotenschwellung, allgemeines Krankheitsgefühl charakteristisch.	E1
23	Symptoms of acute HIV disease.	Symptome einer akuten HIV-Erkrankung.	E5
24	For STI, pain on urination or rash, infection in the genital area would be characteristic.	Für STI wären Schmerzen beim Wasserlassen oder Hautausschlag, Infektionen im Genitalbereich charakteristisch.	E1
25	Alarm symptoms: Fever, rash, visual disturbances, shortness of breath, chest pain, bleeding signs, opportunistic and non-opportunistic.	Alarmsymptome: Fieber, Hautausschlag, Sehstörungen, Luftnot, Brustschmerzen, Blutungszeichen, opportunistisch und nicht-opportunistisch.	E1
26	Recording with the help of a scale would be favorable, as well as frequency and duration.	Erfassung mit Hilfe einer Skala wäre günstig, als auch Häufigkeit und Dauer.	E2
27	(high) fever, shortness of breath due to lung infection, swelling of the lymph nodes, weight loss due to tuberculosis or lymphoma or other tumors.	(hohes) Fieber, Luftnot wegen Lungeninfektion, Lymphknotenschwellungen, Gewichtsverlust wegen Tuberkulose bzw. Lymphom oder anderen Tumoren.	E1
28	Fever and night sweats in combination.	Fieber und Nachtschweiß in Kombination.	E2

Number	Paraphrase (translated into English)	Paraphrase (original German)	Expert Number
29	Symptoms that must be asked about must be named very precisely: Tolerance of medication, gastrointestinal complaints, nausea, aspect of weight development, note patient's comorbidities: Diabetes, CHD or tumour disease, STIs, purulent discharge, ulceration, skin changes, lymph node swelling.	Symptome, die erfragt werden müssen, sehr genau zu benennen: Verträglichkeit der Medikation, gastrointestinale Beschwerden, Übelkeit, Aspekt der Gewichtsentwicklung, Komorbiditäten der Patienten beachten: Diabetes, KHK oder Tumorerkrankung, STIs erfragen, eitriger Ausfluss, Ulkusbildung, Hautveränderungen, Lymphknotenschwellung	E3
30	Persistent fever, signs of meningismus (headache), diarrhea, vomiting.	Anhaltendes Fieber, Zeichen von Meningismus (Kopfschmerzen), Durchfall, Erbrechen.	E5
31	Classic symptoms that can mask depression include: loss of libido, loss of drive, sleep disturbance, headaches, the whole psychosomatic range that indicates depression/anxiety.	Klassische Symptome die eine Depression maskieren können abfragen: Libidoverlust bis hin zu Verlust von Antrieb, Schlafstörung, Kopfschmerzen, den ganzen psychosomatischen Bereich der eine Depression/Angststörung anzeigt.	E7
32	A patient with an anxiety disorder or manifest depression is significantly more vulnerable to acquiring HIV infection.	Ein Patient mit einer Angststörung oder einer manifesten Depression für ist deutlich vulnerabler, gefährdeter eine HIV-Infektion zu bekommen.	E7
33	Advantages of on-demand chat for patients: Quick (within a few hours) answering of acute questions possible, possibly even avoiding a visit to the emergency room or appointment in consultation hours.	Vorteile des On-Demand Chats für Patienten: Schnelle (innerhalb weniger Stunden) Beantwortung von akuten Fragen möglich, eventuell sogar Vermeidung von Besuch der Notaufnahme oder Termin in Sprechstunden.	E2
34	The study participant would like it if the findings, which are collected every 3 months, could be transmitted directly to the PrEP users (but also to all patients) via chat, as their	Der Studienteilnehmer fände es gut, wenn die Befunde, die alle 3 Monate erhoben werden, über den Chat direkt an die PrEP-Nutzer (aber auch allen Patienten) übermittelt werden	E1

Number	Paraphrase (translated into English)	Paraphrase (original German)	Expert Number
	transmission is currently very time-consuming. Open questions could then also be clarified via chat or video telephony.	könnten, da deren Übermittlung aktuell sehr aufwendig ist. Offene Fragen könnten dann auch über Chat oder Videotelefonie geklärt werden.	
35	Displaying the viral load or the CD4 values could perhaps also be helpful for patients in the course of the disease, promote interest in the disease, patients could possibly also gain in security through a positive/stable value course.	Darstellung der Viruslast oder der CD4-Werte könnte vielleicht auch im Verlauf für Patienten hilfreich sein, das Interesse für die Erkrankung fördern; Patienten könnten eventuell durch einen positiven/stabilen Werteverlauf auch an Sicherheit gewinnen.	E5
36	The study participant would like information/findings to be able to be entered into the app by both patients and other caregivers.	Der Studienteilnehmer wünscht sich, dass Informationen/Befunde sowohl von Patienten als auch von anderen Behandelnden in die App eingetragen werden können.	E7
37	Save the complete analytical examination in the app: Findings, blood values, e.g. for another doctor.	Komplette analytische Untersuchung in der App abspeichern: Befunde, Blutwerte z.B. für einen anderen FA.	E7
38	For PrEP, as needed and daily: document and to determine compliance, i.e., review/show tablet use, as this is where errors often occur. Also used as a reminder at re-presentation (e.g., quarterly appointments).	Bei PrEP bedarfsabhängig und täglich: dokumentieren und um die Compliance festzustellen, d.h. Überprüfung/Aufzeigen der Tabletteneinnahme, da dort oft Fehler passieren. Auch als Erinnerungshilfe bei Wiedervorstellung (z. B. bei vierteljährlichen Terminen).	E3
39	Documentation when PreP was interrupted.	Dokumentation wann PreP unterbrochen wurde.	E4
40	The app should not be too complicated, query as few parameters as necessary, only important markers.	Die App darf nicht zu kompliziert sein; so wenige Parameter wie nötig abfragen; nur wichtige Marker	E2

Number	Paraphrase (translated into English)	Paraphrase (original German)	Expert Number
41	Chat cannot/should not replace regular quarterly visits of patients.	Der Chat kann/soll nicht reguläre Quartalsvisite der Patienten ersetzen.	E6
42	It would also be critical that information could not be understood or misinterpreted. Or that fears arise and are not noticed (direct feedback is missing in contrast to the telephone call). App not suitable at the beginning of treatment (sensitive phase, direct contact very important), rather for patients (especially men) who have 'arrived with their diagnosis', to request information in between or to clarify questions quickly.	Kritisch wäre auch, dass Informationen nicht verstanden oder missinterpretiert werden könnten. Oder dass Ängste auftauchen und man das nicht mitbekommt (direktes Feedback fehlt im Gegensatz zum Telefonat). App nicht geeignet zu Beginn der Behandlung (sensible Phase, direkter Kontakt ganz wichtig), eher für Patienten (insbesondere Männer), die „angekommen sind mit ihrer Diagnose", um zwischendrin Informationen abzufragen oder schnell Fragen zu klären.	E8
43	The age group of HIV patients is able to use a smartphone (tends to be younger), no restrictions for specific subgroups (homeless people often have cell phones too, for them, app use would be easier than phone or email).	Die Altersgruppe von HIV-Patienten ist sehr geeignet, um mit einem Handy umzugehen (tendenziell jünger); keine Einschränkungen für bestimmte Teilgruppen (auch Obdachlose haben oft ein Handy, für diese wäre die App-Nutzung einfacher als Telefon oder E-Mail).	E1
44	PrEP users: especially good, mostly younger, tech-savvy - mostly (high) school graduates, grasp it better.	PrEP: besonders gut, meist jünger, technikaffin - meist (Hoch)Schulabschluss, begreifen das besser.	E4
45	The language barrier is a big problem; a multilingual app would be desirable. English/French in the app would not help with this group either.	Die Sprachbarriere ist ein großes Problem, eine mehrsprachige App wäre wünschenswert. Auch Englisch/Französisch in der App würde bei dieser Gruppe nicht weiterhelfen.	E2
46	A multilingual app is very important, the app should include a translation	Eine mehrsprachige App ist sehr wichtig, und würde sich eine App	E7

Number	Paraphrase (translated into English)	Paraphrase (original German)	Expert Number
	feature that facilitates communication and can translate what the patient is talking about live and play back the translation on the screen.	wünschen, die eine Übersetzungsfunktion enthält, die die Kommunikation erleichtert und das Gesprochene des Patienten live übersetzen kann und die Übersetzung auf dem Bildschirm wiedergibt.	
47	Such a tool can be used to care for more patients than is currently possible.	Mit einem solchen Tool kann man mehr Patienten betreuen, als es bisher möglich ist.	E5
48	Ability to communicate with patients when they are not on site and discuss how the year was progressing for them. This would have helped a lot during the COVID period, as some patients came out of fear.	Möglichkeit mit Patienten zu kommunizieren, wenn sie nicht vor Ort sind und besprechen können wie das Jahr verlaufen ist, ob alles gemacht worden ist. Dies hätte in der Corona-Zeit sehr geholfen, da einige Patienten aus Angst gekommen sind.	E7
49	Additional possibility of interaction with patient. Possibility to establish contact with the patient. Especially for patients who would otherwise not contact us.	Zusätzliche Interaktionsmöglichkeit mit dem Patienten. Möglichkeit Kontakt dem mit Patienten aufzubauen. Speziell für Patienten/Patientinnen, die sich sonst nicht melden würden.	E3
50	In addition, unnecessary visits to the outpatient clinic could be avoided, as findings can be discussed directly via video call and prescriptions for medication can be ordered directly.	Zudem könnten nicht notwendige Besuche in der Ambulanz vermieden werden, indem Befunde direkt über die Video-Telefonie besprochen werden können, als auch Rezepte direkt bestellt werden können.	E2
51	Easing bureaucracy, by allowing patients to retrieve findings from app.	Erleichterung der Bürokratie, dadurch, dass Patienten Befunde von App abrufen können.	E6
52	Free up time to maintain the app - 1-2h/week.	Free up time to maintain the app - 1-2h/week.	E6
53	Keep appointment slots free when chat is requested.	Terminslots freihalten, wenn Chat verlangt wird.	E7

Number	Paraphrase (translated into English)	Paraphrase (original German)	Expert Number
54	Keep fixed times.	Feste Zeiten einhalten.	E5
55	Exclusively work computer. No messages on private or work mobile phones desired, as it is very difficult to distinguish between them.	Ausschließlich Arbeitsrechner. Keine Mitteilungen auf privatem Handy oder Diensthandy erwünscht, da Abgrenzung sehr schwierig ist.	E5
56	Use on the private mobile phone is not desired.	Nutzung auf dem Privathandy ist nicht erwünscht.	E2
57	Must be connected to practice/clinic software, with interface to practice/clinic software, must be documented in the practice/clinic system - in the app is not sufficient. Uncertain whether an app can be connected to all existing practice/clinic interfaces.	Muss an Praxis-/Kliniksoftware angebunden sein, mit Schnittstelle zur Praxis-/Kliniksoftware, muss im Praxis-/Kliniksystem dokumentiert werden - in der App reicht nicht aus. Unsicher, ob eine App an alles bisherigen Praxis-/Klinikschnittstellen angebunden werden kann.	E4
