# Supplementary material for: Interviews with HIV Experts for Development of a Mobile Health Application in HIV Care—A Qualitative Study
Source: Healthcare (Basel). 2023 Aug 1;11(15):2180. doi: 10.3390/healthcare11152180 (PMC10418895; doi:10.3390/healthcare11152180)
Supplement: Supplementary file 1 [file healthcare-11-02180-s001.zip › Supp Files/Supplementary File S4.pdf]

User group profiles

Late Presenter

Socio-demographic characteristics	Description
Age	• typically in their late 30s<br>• women: typically, post-menopausal
Gender	• male<br>• female
Homelessness, drug use, refugees/migrants	• migrants screening during asylum application, in reception centers
Residential environment	• urban<br>• rural
Insurance status	• public health insurance<br>• private health insurance
Financial status/income	• often challenging psychosocial and economic circumstances
Distance to treatment center/HIV clinic	• uses app due to distance/transportation issues<br>• partly with long travel distance
Education	• often not well-informed about HIV/AIDS

Task-related characteristics	Description
Experience with mobile phone (Smartphone)/Tablet/Apps	• Young, old, homeless, migrant: has a mobile phone and experience<br>• Elderly: does not have a mobile phone and no experience<br>• HIV app on mobile phone is critical as it may indicate HIV infection
Health literacy	• App educates/raises awareness about symptoms and increases knowledge about the disease<br>• Knowledge about performed tasks (e.g., symptom tracking)<br>• Schedules appointments through the app<br>• Clarifies open questions with the doctor through the app<br>• Records symptoms (psychological, somatic), side effects, and symptom intensity in a symptom diary<br>• Retrieves test results from the app<br>• Checks CD4 count through the app<br>• Enter medications into the app to prevent interactions with other medications prescribed by other healthcare providers
Abilities related to performed tasks (e.g., symptom tracking)	• Is unable to use the app due to the medical condition

Tasks	Subtask
Enable symptom tracking	• Assess disease progression/acute treatment needs<br>• Recognize medication side effects
Facilitate contact	• Contact regarding the occurrence of symptoms<br>• Contact for appointment scheduling/changes<br>• Contact for urgent questions
Enable communication/interaction	• Adjust medication (opportunistic infections, HIV)<br>• Identify emergencies<br>• Transmit findings/test results<br>• Overcome spatial distance<br>• Enable patient-driven interaction

Ressources:

- App, smartphone, tablet, notebook/PC
- Fever thermometer
- Pulse monitor (e.g., smartwatch)

**Organizational characteristics**	**Description**
Time of therapy initiation	• After inpatient treatment or diagnosis of AIDS-defining illness (severe infectious disease, tumor disease such as Kaposi's sarcoma)<br>• Undiagnosed indicator diseases present before HIV diagnosis (e.g., shingles, genital warts, recurrent herpes zoster, recurrent infections, B symptoms, swollen lymph nodes)<br>• HIV test performed at primary care physician<br>• Wants to resume therapy after a break
Therapy course	• Lost-to-follow-up situation
Co-care by primary care physician	• Primary care physician diagnoses HIV<br>• Requires referral from primary care physician (e.g., for insured patients)<br>• Does not require referral from primary care physician (e.g., private patient, HIV expert is also a primary care physician)
Co-care/support by others	• Utilizes services from AIDS assistance organizations, checkpoints<br>• Becomes aware of the app through these organizations<br>• Receives assistance from refugee support worker, possibly social worker (in case of limited German language skills)<br>• Participates in discussion groups (e.g., due to migration background)
Co-care by gynecologist, midwife, pediatrician	• Regular preventive check-ups
Reliability (e.g., regarding appointment adherence)/compliance or adherence	• After getting accustomed to the practice<br>• Receives medication reminder from the app<br>• More frequent follow-up appointments at the beginning of therapy (e.g., every 2 weeks)<br>• Long-term follow-up appointments every three to six months<br>• Well-adjusted long-term patient doesn't require additional app support<br>• Initial phase is particularly sensitive and critical for compliance/adherence<br>• Language barriers can also lead to poor adherence
Ability or willingness for self-management	• Uses the app for a specific/limited period of time

**Psychological and social characteristics**	**Description**
Psychological distress (shock due to diagnosis/anxiety/uncertainty)	• Worries about life<br>• Barrier: has difficulties communicating with doctor<br>• Contacts doctor more frequently via app/phone (e.g., for anxiety disorder)<br>• Concerns about data privacy when using an app<br>• Issue regarding warning messages in critical situations

	• Fear, sometimes panic, due to HIV/AIDS diagnosis<br>• Concern about anonymity when using an app
German language Skills	• Mostly German<br>• Has sufficient German language and writing skills for app usage
English Language Skills	• Does not speak German but knows English (up to 10%)
Neither German or English language skills	• Between 10 to 40 %<br>• Healthcare providers desire the availability of the app in addition to German and English in the following languages:<br>• European: Italian, French, Spanish, Portuguese, Russian/Ukrainian, Romanian, other Eastern European languages<br>• Non-European: Tigrinya (Eritrea) and other African languages (specifically Sub-Saharan Africa), Arabic (specifically Syria, Afghanistan), Vietnamese, Turkish<br>• Arranges their own interpreter (e.g., family members, friends) for medical appointments.
Family setting/living reality	• Family/friends: Women are often not informed about HIV infection.<br>• HIV network: Not available for women.

**Physicial and sensory characteristics**	**Description**
CD4 count	• Has low helper cells at the start of therapy<br>• Has inflammation, kidney and liver, hemoglobin levels
Comorbidities	• Diabetes, coronary heart disease, tumor diseases, STIs
Psychiatric conditions	• depressive mood/depression, suicidal thoughts, anxiety
Medication interactions/side effects	• Experiences in interactions/side effects<br>• Abdominal pain, diarrhea, joint pain due to IRISS (immune reconstitution inflammatory syndrome), strange dreams<br>• Critical combinations:<br>○ e.g., fever and night sweats combined, persistent fever, signs of meningismus, diarrhea, vomiting
Symptoms	• High fever, pulse, chills, diarrhea, rash, nausea, vision problems, chest pain, bleeding signs<br>• Respiratory conditions: cough, shortness of breath, respiratory rate<br>• Tumor diseases: lymph node swelling, night sweats, weight loss<br>• Neurological/neurocognitive disorders (especially in late-stage patients): encephalopathy, forgetfulness, difficulties in daily life<br>• B-symptomatic indicator diseases: recurrent genital herpes, herpes zoster across multiple dermatomes, recurrent infections, bacterial pneumonia, unexplained lymph node swelling, inflammatory diseases (gingivitis, vasculitis)<br>• STIs: purulent discharge, ulcer formation, skin changes, lymph node swelling
Symptom intensity	• Scale (for pain and other discomforts)<br>• Sweating, weight loss (in kg), diarrhea, shortness of breath

PrEP users

Socio-demographic characteristics	Description
Age	Mostly young
Gender	• Mostly men<br>• Women who qualify for PrEP often not identified or unaware
Residential environment	Rural
Education	Mostly high school or higher education

Task-related characteristics	Description
Experience in using mobile phones/smartphones/apps	Technologically inclined
Health literacy	• Possesses knowledge about therapy/diseases (HIV, STIs, etc.)<br>• Contacts doctor due to fear of STIs<br>• App provides education/raises awareness about symptoms
Knowledge about performed tasks	• Schedules appointments through the app<br>• Clarifies questions with doctor through the app<br>• Records symptoms, side effects, symptom intensity in symptom diary<br>• Retrieves findings from the app<br>• Enters medications into the app to prevent interactions with other medications
Motivation related to perform tasks	• (Quick) appointment scheduling through the app<br>• Desires fewer in-person appointments<br>• Pre-orders prescriptions<br>• Uses the app to cope with uncertainties

Tasks	Subtask
Symptom tracking	• Identify acute treatment needs<br>• Identify medication side effects
Contact	• Due to the occurrence of symptoms<br>• For scheduling appointments<br>• Follow-up: every 3 months, or monthly at the beginning<br>• For spontaneous questions
Communication/Interaction	• Transmit findings<br>• Overcome spatial distance<br>• Enable patient-driven interaction

Ressources:

- App, smartphone, tablet, notebook/PC
- Fever thermometer

Organizational characteristics	Description
Co-care by general practitioner (GP)	• Requires referral from GP<br>• Does not require referral from GP<br>• GP prescribes PrEP<br>• Does not have a GP
Initiation of PrEP therapy	• Based on recommendations from GP, other doctors, PrEP users<br>• Based on personal research (e.g., internet)<br>• After consulting GP/HIV experts with primary care involvement
Reliability/compliance	• Takes medication regularly<br>• Uses app as a reminder/diary<br>• Has/attends regular follow-up appointments<br>• STI screenings every 4-12 weeks<br>• Kidney checks

Psychological and social characteristics	Description
Psychological distress	Concerns about data privacy when using an app
German language skills	Speaks good German
English language skills	Up to 20 %
Neither German nor English language skills	• Up to 20 %<br>• Uses the app in languages: Spanish, Portu-guese, Italian, Ukrainian
Sexual orientation/sexual practices	MSM (Men who have sex with men)

Physical and sensory characteristics	Description
Psychiatric conditions	Has anxiety disorder, depression
Acute changes, emerging STIs	• Fever, apathy, discomfort during urination, swol-len lymph nodes<br>• purulent discharge, skin rash/lesions, ulceration, itching, weight loss,<br>• night sweats, pain during defecation
Symptoms of HIV infection	• Persistent diarrhea, fever, skin rash, swollen lymph nodes,<br>• recurrent inflammations
Symptom intensity	Scale, weight loss, symptom yes/no
Side effects of PrEP medications	• Mostly at the beginning of therapy, observed in lab tests<br>• sleep disturbances, concentration problems, daytime sleepiness
Critical constellations	• e.g., fever, signs of inflammation, general feel-ing of illness,<br>• purulent discharge, pain during sexual activity
